# Supplementary material for: Machine Learning Accelerates Crystallization for Structure Determination
Source: Angew Chem Int Ed Engl. 2026 May 4;65(25):e1218503. doi: 10.1002/anie.1218503 (PMC13266958; doi:10.1002/anie.1218503)

checkCIF/PLATON report

Structure factors have been supplied for datablock(s) lcz025

THIS REPORT IS FOR GUIDANCE ONLY. IF USED AS PART OF A REVIEW PROCEDURE FOR PUBLICATION, IT SHOULD NOT REPLACE THE EXPERTISE OF AN EXPERIENCED CRYSTALLOGRAPHIC REFEREE.

No syntax errors found.   CIF dictionary   Interpreting this report

Datablock: lcz025

Bond precision:	C-C = 0.0198 A		Wavelength=1.54184
Cell:	a=12.5404(1)	b=12.0402(1)	c=21.4876(2)
	alpha=90	beta=94.207(1)	gamma=90
Temperature:	100 K		

	Calculated	Reported
Volume	3235.65(5)	3235.65(5)
Space group	C c	C 1 c 1
Hall group	C -2yc	C -2yc
Moiety formula	C15 H3 Ag3 F18 N6, C10 H10 O3	C15 H3 Ag3 F18 N6, C10 H10 O3
Sum formula	C25 H13 Ag3 F18 N6 O3	C25 H13 Ag3 F18 N6 O3
Mr	1111.02	1111.02
Dx,g cm-3	2.281	2.281
Z	4	4
Mu (mm-1)	15.795	15.795
F000	2128.0	2128.0
F000'	2138.43	
h,k,lmax	15,15,27	15,15,27
Nref	6933[3470]	4851
Tmin,Tmax	0.092,0.110	0.692,1.000
Tmin'	0.014	

Correction method= # Reported T Limits: Tmin=0.692 Tmax=1.000
AbsCorr = MULTI-SCAN

Data completeness= 1.40/0.70   Theta(max)= 78.481

R(reflections)= 0.0466(4686)   wR2(reflections)= 0.1213(4851)

S = 1.093   Npar= 498

The following ALERTS were generated. Each ALERT has the format
test-name_ALERT_alert-type_alert-level.
Click on the hyperlinks for more details of the test.

Alert level C

```
PLAT090_ALERT_3_C Poor Data / Parameter Ratio (Zmax > 18) ........       6.69 Note
PLAT213_ALERT_2_C Atom F011            has ADP max/min Ratio .....        3.1 prolat
PLAT213_ALERT_2_C Atom F01J            has ADP max/min Ratio .....        3.1 prolat
PLAT220_ALERT_2_C NonSolvent   Resd 1  F   Ueq(max)/Ueq(min) Range        4.5 Ratio
PLAT342_ALERT_3_C Low Bond Precision on  C-C Bonds ...............    0.01976 Ang.
PLAT911_ALERT_3_C Missing FCF Refl Between Thmin & STh/L=    0.600          7 Report
               -8  0  8,  -1 13 10, -13  5 11,  -1 13 11,  -5  9 16,   3  9 16,
               -5  9 17,
PLAT934_ALERT_3_C Number of (Iobs-Icalc)/Sigma(W) > 10 Outliers ..          1 Check
                1 11-14,
```

Alert level G

```
PLAT003_ALERT_2_G Number of Uiso or U(i,j)  Restrained non-H-Atoms          2 Report
PLAT083_ALERT_2_G SHELXL Second Parameter in WGHT  Unusually Large      64.01 Why ?
PLAT142_ALERT_4_G s.u. on b - Axis Small or Missing .............    0.00010 Ang.
PLAT143_ALERT_4_G s.u. on c - Axis Small or Missing .............    0.00020 Ang.
PLAT186_ALERT_4_G The CIF-Embedded .res File Contains ISOR Records          1 Report
PLAT242_ALERT_2_G Low    'MainMol' Ueq as Compared to Neighbors of       C014 Check
PLAT242_ALERT_2_G Low    'MainMol' Ueq as Compared to Neighbors of       C01E Check
PLAT242_ALERT_2_G Low    'MainMol' Ueq as Compared to Neighbors of       C01F Check
PLAT242_ALERT_2_G Low    'MainMol' Ueq as Compared to Neighbors of       C01G Check
PLAT242_ALERT_2_G Low    'MainMol' Ueq as Compared to Neighbors of       C01I Check
PLAT434_ALERT_2_G Short Inter HL..HL Contact F005     ..F00S     .       2.80 Ang.
                                  -1/2+x,1/2-y,1/2+z  =      4_455 Check
PLAT720_ALERT_4_G Number of Unusual/Non-Standard Labels ..........         67 Note
              Ag01    Ag02    Ag03    F004    F005    F006    F007    F008
              N009    F00A    F00B    N00C    O00D    N00E    N00F    F00G
              F00H    F00I    N00J    O00K    O00L    F00M    F00N    F00O
              C00Q    H00Q    C00R    H00A    H00B    F00S    F00T    C00U
              C00V    C00W    C00X    F00Y    C00Z    H00Z    C010    F011
              C012    H012    C013    H013    C014    C015    C016    C017
              H017    C018    H018    C019    C01A    C01B    C01C    H01A
              H01B    C01D    C01E    C01F    C01G    C01H    H01C    H01D
              H01E    C01I    F01J
PLAT860_ALERT_3_G Number of Least-Squares Restraints ............         14 Note
PLAT883_ALERT_1_G Absent Datum for _atom_sites_solution_primary ..     Please Do !
PLAT910_ALERT_3_G Missing FCF Reflection(s) Below Theta(Min)[Deg]=       5.10 Note
                0  0  2,
PLAT912_ALERT_4_G Missing # of FCF Reflections Above STh/L=  0.600        131 Note
PLAT915_ALERT_3_G No Flack x Check Done: Low Friedel Pair Coverage         44 %
PLAT941_ALERT_3_G Average HKL Measurement Multiplicity ..........          2.9 Low
```

```
PLAT969_ALERT_5_G The 'Henn et al.' R-Factor-gap value ..........      3.511 Note
              Predicted wR2: Based on SigI**2  3.46 or SHELX Weight 11.11
PLAT978_ALERT_2_G Number C-C Bonds with Positive Residual Density.         0 Info
```

```
   0 ALERT level A = Most likely a serious problem - resolve or explain
   0 ALERT level B = A potentially serious problem, consider carefully
   7 ALERT level C = Check. Ensure it is not caused by an omission or oversight
  20 ALERT level G = General information/check it is not something unexpected

   1 ALERT type 1 CIF construction/syntax error, inconsistent or missing data
  12 ALERT type 2 Indicator that the structure model may be wrong or deficient
   8 ALERT type 3 Indicator that the structure quality may be low
   5 ALERT type 4 Improvement, methodology, query or suggestion
   1 ALERT type 5 Informative message, check
```

Validation response form

Please find below a validation response form (VRF) that can be filled in and pasted into your CIF.

```
# start Validation Reply Form
_vrf_PLAT090_lcz025
;
PROBLEM: Poor Data / Parameter Ratio (Zmax > 18) ........       6.69 Note
RESPONSE: ...
;
_vrf_PLAT213_lcz025
;
PROBLEM: Atom F011            has ADP max/min Ratio .....        3.1 prolat
RESPONSE: ...
;
_vrf_PLAT220_lcz025
;
PROBLEM: NonSolvent   Resd 1  F   Ueq(max)/Ueq(min) Range        4.5 Ratio
RESPONSE: ...
;
_vrf_PLAT342_lcz025
;
PROBLEM: Low Bond Precision on  C-C Bonds ..............    0.01976 Ang.
RESPONSE: ...
;
_vrf_PLAT911_lcz025
;
PROBLEM: Missing FCF Refl Between Thmin & STh/L=    0.600          7 Report
RESPONSE: ...
;
_vrf_PLAT934_lcz025
;
PROBLEM: Number of (Iobs-Icalc)/Sigma(W) > 10 Outliers ..          1 Check
RESPONSE: ...
;
# end Validation Reply Form
```

It is advisable to attempt to resolve as many as possible of the alerts in all categories. Often the minor alerts point to easily fixed oversights, errors and omissions in your CIF or refinement strategy, so attention to these fine details can be worthwhile. It is up to the individual to critically assess their own results and, if necessary, seek expert advice.

PLATON version of 17/09/2025; check.def file version of 17/09/2025

---

duplicate check

No duplication found

Datablock lcz025 - ellipsoid plot

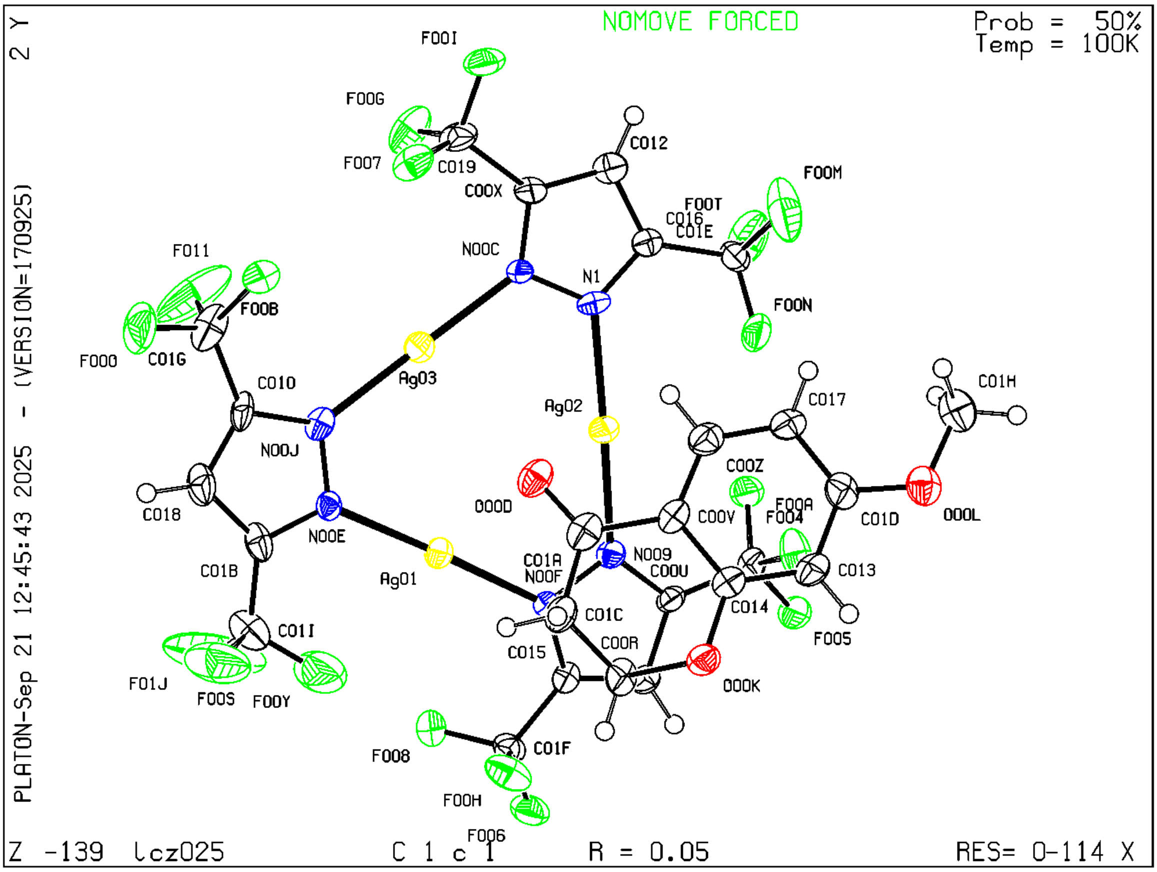

Supplement: Supplementary file 2 — Supporting File 2: anie72462‐sup‐0002‐DataFile.zip. [file ANIE-65-e1218503-s002.zip › supplementary_materials_crystallographic_data submit.zip!/cif_and_checkcif(1-114)/Ag3Pz3_25/Ag3Pz3_25_checkcif.pdf]
